# Phyllodes Tumor of Breast: A Review Article

**DOI:** 10.1155/2013/361469

**Published:** 2013-03-20

**Authors:** Shashi Prakash Mishra, Satyendra Kumar Tiwary, Manjaree Mishra, Ajay Kumar Khanna

**Affiliations:** Department of Surgery, Institute of Medical Sciences, Banaras Hindu University, Varanasi, Ultar Pradesh 221005, India

## Abstract

*Introduction*. Phyllodes tumours are rare fibroepithelial lesions. Accurate preoperative pathological diagnosis allows correct surgical planning and avoidance of reoperation. Treatment can be either wide local excision or mastectomy to achieve histologically clear margins. *Discussion*. The exact aetiology of phyllodes tumour and its relationship with fibroadenoma are unclear. Women aged between 35 and 55 years are commonly involved. The median tumour size is 4 cm but can grow even larger having dilated veins and a blue discoloration over skin. Palpable axillary lymphadenopathy can be identified in up to 10–15% of patients but <1% had pathological positive nodes. Mammography and ultrasonography are main imaging modalities. Cytologically the presence of both epithelial and stromal elements supports the diagnosis. The value of FNAC in diagnosis of phyllodes tumour remains controversial, but core needle biopsy has high sensitivity and negative predictive value. Surgical management is the mainstay and local recurrence in phyllodes tumours has been associated with inadequate local excision. The role of adjuvant radiotherapy and chemotherapy remains uncertain and use of hormonal therapy has not been fully investigated. *Conclusion*. The preoperative diagnosis and proper management are crucial in phyllodes tumours because of their tendency to recur and malignant potential in some of these tumours.

## 1. Introduction

Phyllodes tumors are rare fibroepithelial lesions. They make up 0.3 to 0.5% of female breast tumors [1] and have an incidence of about 2.1 per million, the peak of which occurs in women aged 45 to 49 years [2, 3]. The tumor is rarely found in adolescents and the elderly. They have been described as early as 1774, as a giant type of fibroadenoma [4]. Chelius [5] in 1827 first described this tumor. *Johannes Muller (1838)* was the first person to use the term *cystosarcoma phyllodes. *It was believed to be benign until 1943, when* Cooper and Ackerman *reported on the malignant biological potential of this tumor. In *1981 [6] the World Health Organization* adopted the term phyllodes tumor and as described by Rosen [7] subclassified them histologically as benign, borderline, or malignant according to the features such as tumor margins, stromal overgrowth, tumor necrosis, cellular atypia, and number of mitosis per high power field. The majority of phyllodes tumors have been described as benign (35% to 64%), with the remainder divided between the borderline and malignant subtypes. The term phyllodes tumor represents a broad range of fibroepithelial diseases and presence of an epithelial component with stromal components differentiates the phyllodes tumor from other stromal sarcomas.

Accurate preoperative pathological diagnosis allows correct surgical planning and avoidance of reoperation, either to achieve wider excision or for subsequent tumor recurrence [8–10]. At one extreme, malignant phyllodes tumors, if inadequately treated, have a propensity for rapid growth and metastatic spread. In contrast, benign phyllodes tumors on clinical, radiological, and cytological examination are often indistinguishable from fibroadenomas and can be cured by local surgery. With the nonoperative management of fibroadenomas widely adopted, the importance of phyllodes tumors today lies in the need to differentiate them from other benign breast lesions. Treatment can be either wide local excision or mastectomy provided histologically clear specimen margins are ensured [2, 11, 12].

## 2. Etiology

At present time, the exact etiology of phyllodes tumor and its relationship with fibroadenoma are unclear. Noguchi et al. [13] showed that most fibroadenomas have polyclonal elements and should be regarded as hyperplasic rather than neoplastic lesions. It has been suggested that, in a proportion of fibroadenomas, a somatic mutation can result in a monoclonal proliferation, histologically indistinguishable from the polyclonal element, but with a propensity to local recurrence and progression to a phyllodes tumor which has also been supported by clonal analysis. It has also been postulated that stromal induction of phyllodes tumors can occur as a result of growth factors produced by the breast epithelium. Trauma, lactation, pregnancy, and increased estrogen activity occasionally have been implicated as factors stimulating tumor growth. The nature of these factors is unclear but endothelin-1, a stimulator of breast fibroblast growth, may be important.

## 3. Pathogenesis

Unlike carcinoma breast, phyllodes tumors start outside of the ducts and lobules, in the breast's connective tissue, called the stroma which includes the fatty tissue and ligaments that surround the ducts, lobules, and blood and lymph vessels in the breast. In addition to stromal cells, phyllodes tumors can also contain cells from the ducts and lobules.

## 4. Classification

### 4.1. WHO Criteria

World Health Organization divided phyllodes tumor into benign, borderline, and malignant categories based on the degree of stromal cellular atypia, mitotic activity per 10 high power fields, degree of stromal overgrowth (these three are main), tumor necrosis, and margin appearance (see Table 1). 

### 4.2. Criteria Proposed by Azzopardi et al. [14] and Salvadori et al. [2]

See Table 2.

## 5. Diagnosis

### 5.1. Clinical Presentation

Most of the tumor arises in women aged between 35 and 55 years (approximately 20 years later than fibroadenoma), 3, 15, 16 more prevalent in the Latin American white and Asian populations [3]. Few cases have been reported in men and these have invariably been associated with the presence of gynaecomastia. It usually presents as a rapidly growing but clinically benign breast lump. In some patients a lesion may have been apparent for several years, with clinical presentation precipitated by a sudden increase in size [15, 16].

**Figure 1:**
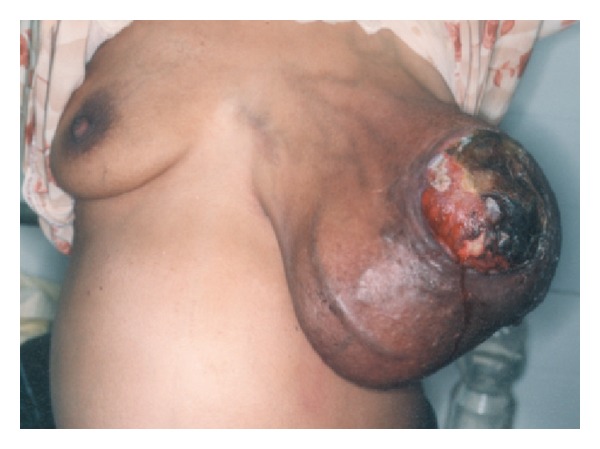
Giant phyllodes tumor.

- The skin over large tumors may have dilated veins and a blue discoloration but nipple retraction is rare.
- Fixation to skin and pectoralis muscles has been reported, but ulceration is uncommon.
- More commonly found in upper outer quadrant with an equal propensity to occur in either breast.
- Rarely presentation may be bilateral.
- The median size of phyllodes tumors is around 4 cm. 20% of tumors grow larger than 10 cm (giant phyllodes tumor). These tumors can reach sizes up to 40 cm in diameter (see Figure 1).
- A significant proportion of patients have history of fibroadenoma and in a minority these have been multiple.
- Palpable axillary lymphadenopathy can be identified in up to 10–15% of patients but <1% had pathological positive nodes.

### 5.2. Radiological Investigations

Mammography and ultrasonography are mainstay of routine imaging of breast lumps. Wurdinger et al. [17] show that round or lobulated shape, well-defined margins, heterogeneous internal structure, and nonenhancing internal septations are more common findings in phyllodes tumors than in fibroadenomas. 

#### 5.2.1. Ultrasonography [18, 19]

 Lobulated shape (in some cases round or oval) well circumscribed with smooth margins, echogenic rim, and low level homogenous internal echoes. Fluid-filled clefts in a predominantly solid mass (highly suggestive of phyllodes tumor) with good thorough transmission and lack of microcalcification are seen. 

#### 5.2.2. Color Doppler Ultrasonography

Features suggesting malignant behavior are

- marked hypoechogenicity,
- posterior acoustic shadowing,
- ill-defined tumor margins.
- higher values of RI (resistance index),
- increased PI (pulsatility index),
- increased Vmax (systolic peak flow velocity).

#### 5.2.3. Mammography [18, 20, 21] (see Figure 2)

- It shows well circumscribed oval or lobulated mass with rounded borders.
- A radiolucent halo may be seen around the lesion due to compression of the surroundings.
- Coarse calcification (but malignant microcalcification is rare) may be present.

#### 5.2.4. Magnetic Resonance Imaging (MRI) [21–28]

The following features are mainly found in phyllodes tumor on MRI:

- round or lobulated shape and well-defined margins,
- heterogeneous internal structure/nonenhancing septations,
- exhibits hypointense signals on T1-weighted images,
- exhibits hyper/isointense signals on T2-weighted images,
- contrast enhancement pattern:
  - benign lesion:
    - slow initial enhancement with persistent delayed phase;
  - malignant lesion:
    - fast initial enhancement with plateau phage,
    - fast initial enhancement with wash-out phenomenon.

### 5.3. Pathological/Histological Assessment

As both phyllodes tumors and fibroadenomas belong to a spectrum of fibroepithelial lesions, accurate cytological diagnosis of phyllodes tumors by fine needle aspiration can be difficult.

Cytologically, it is often easier to differentiate benign from malignant phyllodes tumors than to separate benign phyllodes tumors from fibroadenomas. In the correct clinical setting, the presence of both epithelial and stromal elements within the cytological smear supports the diagnosis. Epithelial cells may, however, be absent from specimens taken from malignant lesions. The presence of cohesive stromal cells (phyllodes fragments), isolated mesenchymal cells, clusters of hyperplastic duct cells, foreign body giant cells, blood vessels crossing the stromal fragments, and bipolar naked nuclei and the absence of apocrine metaplasia are highly suggestive of a phyllodes tumor. However, the value of FNAC in the diagnosis of phyllodes tumor remains controversial, with an overall accuracy of about 63% [29, 30]. Core tissue biopsy is an attractive alternative to FNAC because of the extra architectural information provided by histology compared with cytology. Komenaka et al. [31] found the sensitivity of core needle biopsy to be 99% and negative predictive value and positive predictive value 93% and 83%, respectively, for the diagnosis.

#### 5.3.1. Macroscopic Appearance

Macroscopically most small tumors have a uniform white consistency with a lobulated surface, similar to that of a fibroadenoma. Large tumors on cut section often have a red or grey “meaty” consistency with fibrogelatinous, hemorrhagic, and necrotic areas with leaf like protrusions into the cystic spaces.

#### 5.3.2. Microscopic Appearance (see Figures 3–6)

*Fine Needle Aspiration Cytology*. The cytological diagnosis of phyllodes tumors is mainly suggested by the presence of hypercellular stroma and the stromal elements on the smears being more numerous than the epithelial ones. The cells on the smears were classified by Deen et al. [32] in 1999, and Jayaram and Sthaneshwar in 2002 [33], by comparison with small lymphocytes, in

1. short, round/oval cells, two-size smaller than the size of a lymphocyte: considered to be epithelial cells;
2. long, spindle cells, three-size larger than the size of a lymphocyte: considered to be stromal cells.

Many authors considered that the following aspects should also be taken into consideration in the case of cytological diagnosis of phyllodes tumors:

- the presence of hypercellular stromal fragments;
- the cellular composition of the stromal fragments;
- the amount of naked nuclei on the background of the smears;
- the morphology of the naked nuclei (especially the bipolar ones).

See Table 3. 

*Core Needle Biopsy.* Fibroepithelial lesions with cellular stroma in breast core needle biopsy (CNB) specimens may result in either fibroadenoma or phyllodes tumor at excision. Assessment of stromal cellularity, stromal cell atypia, mitoses, and relative proportion of stroma to epithelium are mainly helpful to reach the diagnosis. Phyllodes tumors are usually differentiated histologically from fibroadenoma by its increased stromal cellularity and mitotic activity. However, benign phyllodes tumor by definition lacks marked atypia and excess mitotic activity in its stromal component, and juvenile fibroadenoma may also have cellular stroma, presenting a source of increased diagnostic difficulty. Diagnosis relies on the recognition of the exaggerated intracanalicular growth pattern in phyllodes tumor. In addition, the stromal proliferation in juvenile fibroadenoma tends to be relatively uniform, whereas in phyllodes tumor it is often (though not always) more prominent in the periductal areas. The stromal cellularity in phyllodes tumor may be heterogeneous. Consequently, surgical excision is recommended for complete evaluation of the lesion.

Jacobs et al. [34] found that 4 stromal features in CNB specimens (i.e., cellularity, nuclear atypia, mitoses, and amount of stroma relative to epithelium) differed significantly between cases that were fibroadenoma at excision compared with those that were phyllodes tumor. However, only cases that had mildly or markedly increased stromal cellularity in CNB specimens were absolutely predictive of fibroadenoma or phyllodes tumor, respectively. Among the subset of cases with moderate stromal cellularity in the CNB specimens, the presence of stromal mitoses remained the single histological feature significantly different between the phyllodes tumor and fibroadenoma groups.

Sarcomatous stromal elements, including angiosarcoma, chondrosarcoma, leiomyosarcoma, osteosarcoma, liposarcoma, and rhabdomyosarcoma, are rarely encountered in malignant phyllodes tumors.

*Paddington Clinicopathological Suspicion Score*. This outlines criteria to assist in the selection of patients for core biopsy, for use in conjunction with existing local protocols. The aim of developing the score is to improve the rates of preoperative diagnosis (see Box 1).

#### 5.3.3. Differential Diagnosis

It includes the following:

- fibroadenoma,
- adenoma,
- hamartoma,
- lipoma,
- juvenile papillomatosis,
- carcinoma,
- sarcomas,
- metastatic tumor.

*Management*. Surgical management is the mainstay but the type of surgery has been a source of debate over the years. Studies have shown no differences between breast conserving surgery versus mastectomy in terms of metastasis-free survival or overall survival, despite the higher incidence of local recurrence that comes with breast conserving surgery [35].

If diagnosed preoperatively, tumor should be resected with at least 1 cm margins particularly in the borderline and malignant phyllodes tumors. This can be accomplished by either lumpectomy or mastectomy, depending upon the size of the tumor relative to the breast. For benign phyllodes tumors diagnosed after local excision of what appeared to be a fibroadenoma, a “watch and wait” policy does appear to be safe. With such an approach, local recurrence and five year survival rates of 4% and 96% respectively have been reported for benign phyllodes tumors. Whether patients with benign phyllodes tumors who have undergone local excision and have histologically positive specimen margins should undergo further surgery or be entered in a surveillance program is controversial. Reexcision of borderline and malignant phyllodes tumors identified after local excision should be considered. 

Twenty percent of tumors grow larger than 10 cm, the arbitrary cutoff point for the designation as giant phyllodes tumor, an entity that presents the surgeon with several unique management problems. These tumors can reach sizes up to 40 cm in diameter [15]. Since an excision with the required margins is often impossible in giant phyllodes tumors, mastectomy should be reserved for larger tumors and should be considered in recurrent tumors, especially of the malignant histotype [2, 36–38]. Local recurrence in phyllodes tumors has been associated with inadequate local excision and various histological characteristics, including mitotic activity, tumor margin, and stromal cellular atypia. Depending on the size of the breast and the location of the phyllodes tumor, mastectomy may also be required for tumors that are between 5 and 10 cm in diameter [39]. While managing a giant phyllodes tumor, emphasis should be on complete extirpation of all visible tumors and breast tissue during mastectomy to minimize the chances of recurrence.

As malignant phyllodes tumors undergo mainly hematogenous spread, the proportion of patients with lymph node metastases are <1% (lymph node enlargement in about 10%) and routine axillary clearance is not recommended. Axillary dissection is required, when histologically positive for malignant cells.

Chest wall invasion appears to be an uncommon event with phyllodes tumors [15], extended excision of involved pectoralis muscle, followed by reconstruction of the chest wall with marlex mesh or latissimus dorsi muscular/myocutaneous flap been recommended if the fascia or muscle is infiltrated. Some have recommended the consideration of postoperative radiation for cases of chest wall infiltration.

Foreknowledge of the location of the tumor's blood supply can be vital information when removing large tumors. Jonsson and Libshitz documented the angiographic pattern of a 25 cm phyllodes tumor via one large and several smaller perforating anterior branches of the internal mammary, lateral thoracic, acromiothoracic arteries, and branches of the axillary artery [40]. Liang et al. [41] found that the giant tumors in the present report derived the majority of their blood supply from skin collaterals. Thus, the surgeon can expect the majority of blood loss during resection to come from the creation of the skin flaps. In this situation, the surgeon need not routinely obtain an angiogram.

 In general, immediate breast reconstruction can be performed at the time of mastectomy for phyllodes tumors [42]. Mendel et al. [43] reported a case in which subcutaneous mastectomy was performed for a large phyllodes tumor, followed by immediate implantation of a breast prosthesis. They cite minimal interference with the detection of recurrent lesions and the minimization of emotional distress as advantages to the procedure. Orenstein and Tsur described a similar case in an adolescent female in which a silicon implant was placed under the pectoralis major, where it would not impair the recognition of recurrent disease [44]. Local recurrence rates for phyllodes tumors are 15 to 20% and are correlated with positive excision margins, rather than with tumor grade or size [1, 16, 42, 45]. Other studies have shown a higher risk of local recurrence in borderline and malignant tumors. In a series of 21 patients by Salvadori et al., 51 patients were treated with breast conserving surgery (enucleations, wide excisions), and 14 of the tumors recurred locally. In contrast, the 20 patients treated with mastectomy (subcutaneous, modified radical, or radical) showed no evidence of local recurrence [2]. Importantly, there is no contraindication to immediate reconstruction after mastectomy in cases of giant phyllodes tumor, and this decision can be made solely based upon patient preference [43, 44].

**Figure 7:**
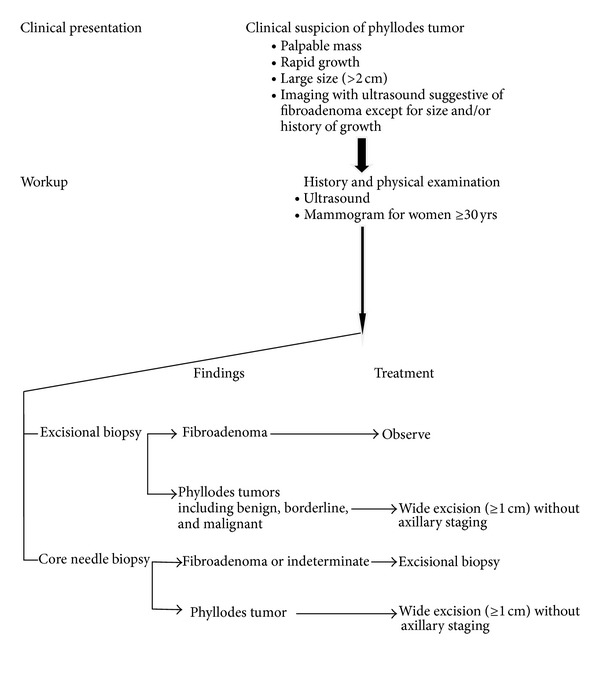


*NCCN Guidelines for the Management of Phyllodes Tumor*. According to NCCN guidelines wide excision means excision with the intention of obtaining surgical margins ≥1 cm. Narrow surgical margins are associated with high local recurrence risk, but are not an absolute indication for mastectomy when partial mastectomy fails to achieve margin width ≥1 cm (see Figure 7).

*Role of Adjuvant Therapy*. The role of adjuvant radiotherapy and chemotherapy remains uncertain, but encouraging results using radiotherapy and chemotherapy for soft-tissue sarcomas suggest that consideration be given for their use in cases of malignant phyllodes tumors [46–50].

Chaney et al. [51] found adjuvant radiotherapy to be beneficial in patients with adverse features (e.g., bulky tumors, close or positive surgical margins, hypercellular stroma, high nuclear pleomorphism, high mitotic rate, presence of necrosis, and increased vascularity within the tumor and tumor recurrence) but the use is controversial. A study done by Richard J. Barth Jr demonstrated that margin-negative resection combined with adjuvant radiotherapy is an effective therapy for local control of borderline and malignant phyllodes tumors. In MD Anderson Cancer Center, radiotherapy is recommended only for cases with positive or near-positive surgical margins and selected cases for whom further surgical procedures cannot be performed.

Chemotherapy, including anthracyclines, ifosfamide, cisplatin, and etoposide, has been mentioned in various studies but with no survival advantage. 

The use of hormonal therapy, such as tamoxifen, has not been fully investigated in cystosarcoma phyllodes. Estrogen and progesterone receptor expression has been shown in 43% and 84%, respectively, of the epithelium and less than 5% of the stromal cells. Still, the use of endocrine therapy in either the adjuvant or palliative setting has not been extensively studied.

*Prognostic Factors*. No reliable clinical prognostic factors have been identified that predict for local recurrence or metastasis. Patient age does not appear to be important but tumors presenting in adolescence do seem to be less aggressive irrespective of their histological type. The size of the tumor not as such but in relation to the breast appears important as this usually determines the extent of surgery and the resulting specimen resection margins. 

Most distant metastases develop from borderline or malignant tumors. Unlike local recurrence, tumor size does appear to be an important factor in predicting for metastatic spread. Many histological prognostic factors have been evaluated. Different studies have regarded stromal overgrowth, tumor necrosis, infiltrating margins, mixed mesenchymal components, high mitotic rate, and stromal atypia as important but in isolation each appears to have a low predictive value.

#### 5.3.4. Role of Tumor Markers in Phyllodes Tumor

Increased p53 protein and Ki-67 antigen expression has been detected in malignant phyllodes tumors and they may be valuable in differentiating fibroadenomas from phyllodes tumors. Furthermore, in phyllodes tumors, p53 and Ki-67 expression has been shown to correlate with negative prognostic factors.

Philip C. W. et al. showed the role of angiogenesis and found that the higher the microvessel density, the higher the degree of malignancy for the phyllodes tumor. 

Gary M. K. Tse et al. found that CD117 protein expression by stromal cells in phyllodes tumors is correlated with histological parameters such as grade, implying a possible role of their being used as adjunctive markers of malignancy in these tumors. In malignant phyllodes tumors, the rate of expression is up to 46%. This provides additional strong evidence that c-kit receptor-mediated tyrosine kinase activity may be involved early on in the pathogenesis of phyllodes tumors, and the new therapeutic agent, STI571, Glivec, may be a useful drug therapy for this disease, particularly in the tumor recurrences and advanced-stage disease. Marick Lae et al. showed that chromosomal changes detected by comparative genomic hybridization (CGH) could be helpful in grading phyllodes tumors. In flow cytometric studies, correlations between DNA content, cell proliferation, and histological grade have been demonstrated. Some studies have identified a correlation between these markers of cellular proliferation and clinical outcome, however, most have not. Recent small studies have suggested that telomerase, a ribonucleoprotein enzyme that generates telomeres (DNA sequences important in determining cell immortality), may be a useful prognostic factor in phyllodes tumors.

*Recurrence.* To date, local recurrence rates ranging from 10% to 40% have been reported with most series averaging about 15%. Local recurrence appears to be related to the extent of the initial surgery and should be regarded as a failure of primary surgical treatment. Whether malignant tumors have an increased risk of recurrence is unclear but when it does occur it is invariably seen earlier than with benign tumors. In multivariate analysis, the surgical margin is found to be the only independent predictive factor for local recurrence. In most patients, local recurrence is isolated and is not associated with the development of distant metastases.

In a minority of patients repeated local recurrence occurs. This is often seen irrespective of either the histological type of the tumor or the extent of the specimen margins. Local recurrence can usually be controlled by further wide excision (with 1 cm margins) and mastectomy is not invariably required. Mastectomy should, however, be considered for local recurrence after local surgery for borderline or malignant tumors. Occasionally aggressive local recurrence can result in widespread chest wall disease with direct invasion of the underlying lung parenchyma. Isolated reports of good palliation in this situation with radiotherapy have been published.

**Figure 8:**
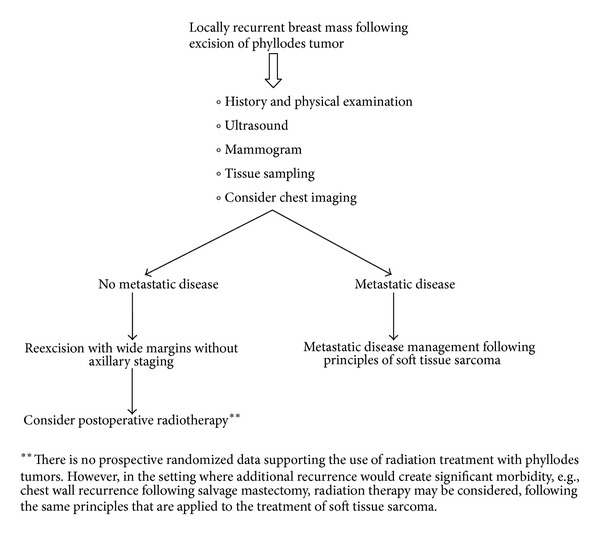


*NCCN Guidelines for the Management of Recurrence.* See Figure 8.

*Metastasis*. Overall, 10% of patients with phyllodes tumors develop distant metastases and these eventually occur in approximately 25% of patients with histologically malignant tumors. Most distant metastases develop without evidence of local recurrence. The commonest sites for distant metastases are the lungs (66%), bones (28%), and brain (9%) and in rare instances, the liver and heart. The risk of metastatic disease does not appear to be influenced by the extent of the initial surgery and appears to be predetermined by tumor biology. Metastatic phyllodes tumors have a poor prognosis and no long-term survival.

*Followup*. Since phyllodes tumors are locally recurrent tumor especially when not excised with a clear margins and very unpredictable in growth and metastatic activity, it is very necessary to follow up the patient regularly at 6-month interval for the first two years (chances of recurrence are maximum in the first two years) and then on yearly basis. Patients must be instructed to self examine her breast regularly and consult her doctor, if any abnormality detected. In followup, patient should be examined and, if any abnormality detected, it should be investigated with USG, mammogram, MRI, or tissue biopsy.

## 6. Conclusion

Phyllodes tumor bears specific clinical characteristic and can be considered as a differential diagnosis for the breast lumps. The preoperative diagnosis and proper management are crucial in phyllodes tumor because of their tendency to recur and malignant potential in some of these tumors.

## Figures and Tables

**Figure 2:**
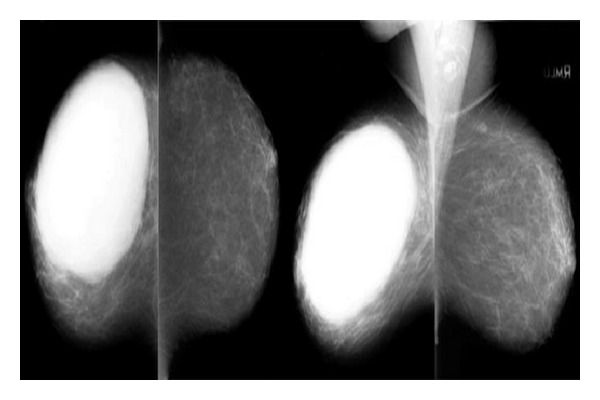
Phyllodes tumor on mammography.

**Figure 3:**
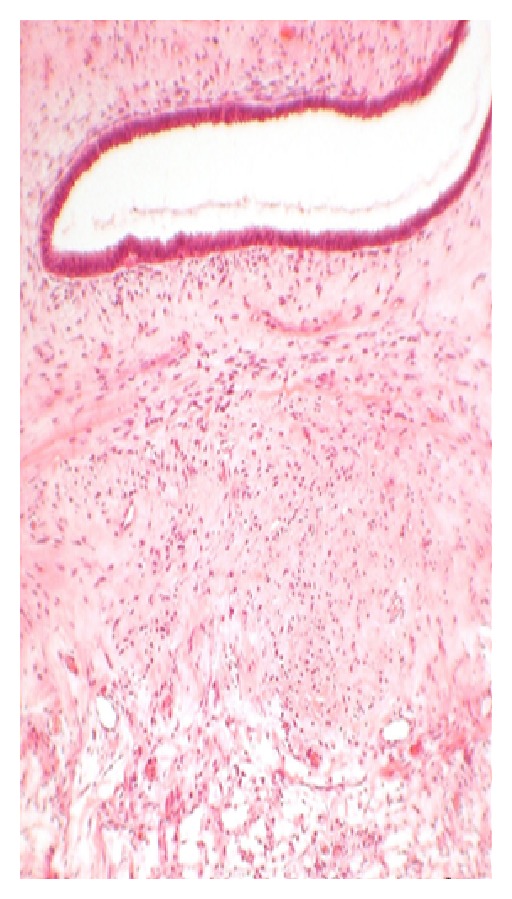
Stained slide showing microphotograph of phyllodes tumor.

**Figure 4:**
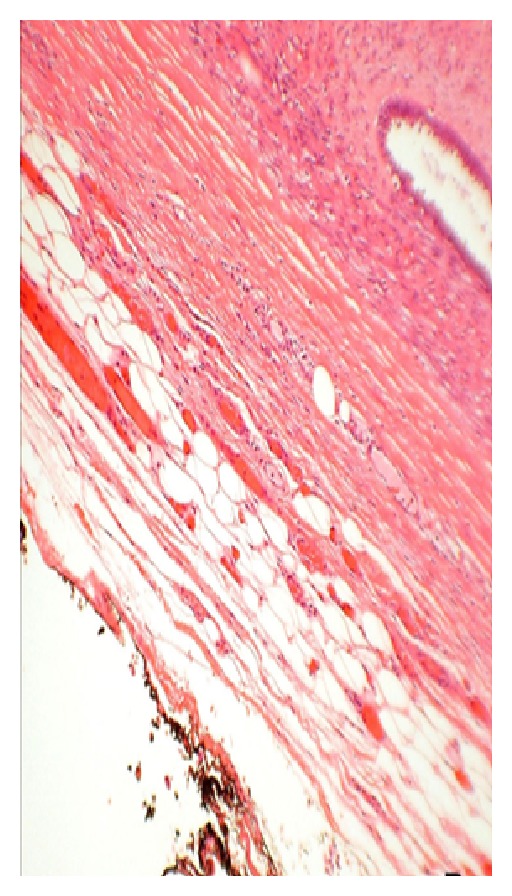
Stained slide showing microphotograph of phyllodes tumor.

**Figure 5:**
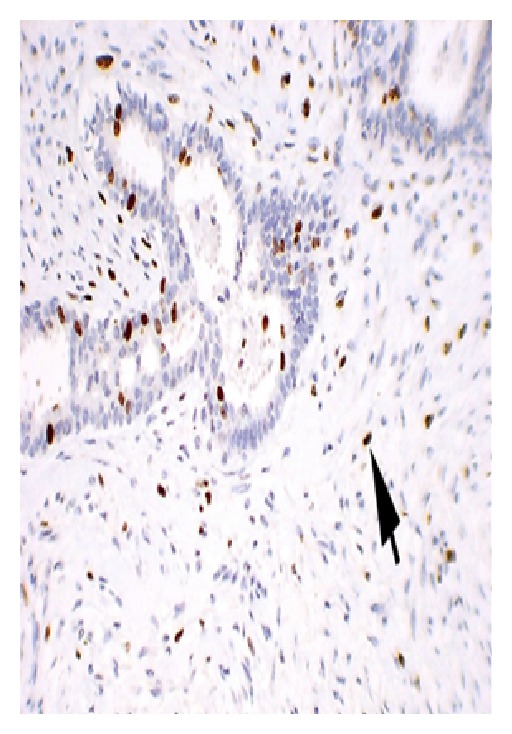
Stained slide showing microphotograph of phyllodes tumor.

**Figure 6:**
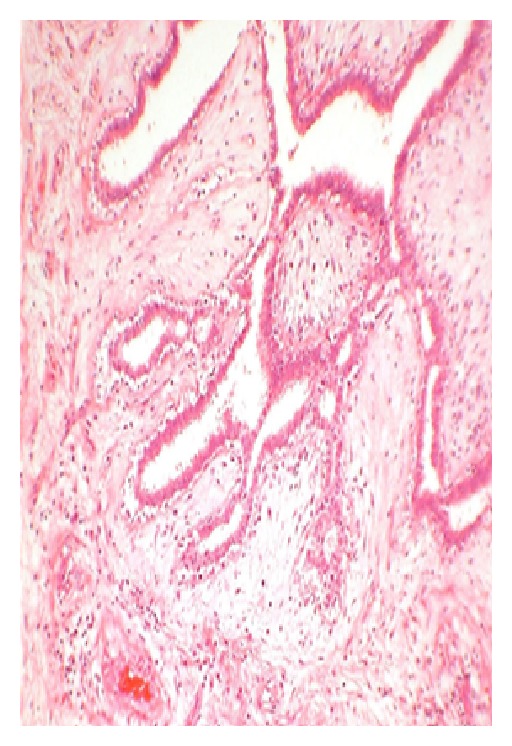
Stained slide showing microphotograph of phyllodes tumor.

**Box 1:**
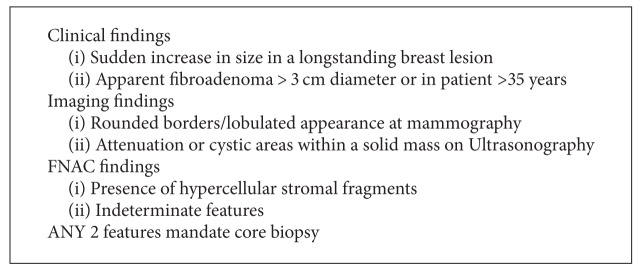


**Table 1:** 

Criteria	Benign	Borderline	Malignant
Stromal cellularity and atypia	Minimal	Moderate	Marked
Stromal overgrowth	Minimal	Moderate	Marked
Mitoses/10 high power fields	0–4	5–9	≥10
Tumor margins	Well circumscribed with pushing tumor margins	Zone of microscopic invasion around tumor margins	Infiltrative tumor margins

**Table 2:** 

Criteria	Histological type		
	Benign	Borderline	Malignant
Tumor margins	Pushing	*↔*	Infiltrative
Stroma cellularity	Low	Moderate	High
Mitotic rate (per 10 hpf)	<5	5–9	≥10
Pleomorphism	Mild	Moderate	Severe

**Table 3:** 

Benign phyllodes tumors	Borderline phyllodes tumors	Malignant phyllodes tumors
The stromal compound, represented by stromal fragments, isolated stromal cells, and naked stromal nuclei are found to be more numerous than the epithelial one in most of the cases.	(i) There is predominance of the stromal component as compared to the epithelial one. (ii) Frequent hypercellular stromal fragments, an average of 2 in each microscopical field. (iii) Frequent large spindle cells and monomorphic naked stromal nuclei.	(i) Stromal fragments of variable dimensions, with moderate cellularity, made of discohesive spindle cells, with atypical nuclei. (ii) Minimal/no epithelial elements found on the smears. (iii) Presence of atypical multinucleated giant cells.
